# Evaluation of Clinical Variables Associated with Increased Carotid Intima-Media Thickness in Middle-Aged Hypertensive Women

**DOI:** 10.1155/2012/257501

**Published:** 2012-06-03

**Authors:** Michelle Trindade, Renata Brum Martucci, Adriana K. Burlá, Wille Oigman, Mario Fritsch Neves, Denizar Vianna Araújo

**Affiliations:** ^1^Department of Clinical Medicine, State University of Rio de Janeiro, Rua Vinte e Oito de Setembro, 77, Sala 329—Vila Isabel, 20551-030—Rio de Janeiro, RJ, Brazil; ^2^Institution of Nutrition, State University of Rio de Janeiro, Rua Vinte e Oito de Setembro, 77, Sala 329, Vila Isabel, 20551-030 Rio de Janeiro, RJ, Brazil

## Abstract

It has been previously documented that carotid intima-media thickness (cIMT) is a predictor of cardiovascular disease. The aim of this study was to identify clinical parameters associated with an increased cIMT treated hypertensive women. Female patients (*n* = 116) with essential hypertension, aged 40–65 years, were included in this study. Vascular ultrasound was performed and the patients were divided into two groups according to the values of cIMT (< or ≥0.9 mm). Patients with greater cIMT presented significantly higher systolic blood pressure and pulse pressure. Serum HDL-cholesterol was significantly lower and CRP was significantly higher in the same group. There was a significant correlation between cIMT and age (*r* = 0.25, *P* = 0.007), systolic blood pressure (*r* = 0.19, *P* = 0.009), pulse pressure (*r* = 0.30, *P* = 0.001), and LDL-cholesterol (*r* = 0.19, *P* = 0.043). cIMT was correlated to CRP (*r* = 0.31, *P* = 0.007) and negatively correlated to HDL-cholesterol (*r* = 0.33, *P* = 0.001). In logistic regression, only HDL-cholesterol, CRP, and pulse pressure were shown to be independent variables associated to increased cIMT. In conclusion, pulse pressure, HDL-cholesterol, and CRP are variables correlated with cIMT in treated hypertensive women.

## 1. Introduction

Atherosclerosis and its complications are the main cause of morbidity and mortality in the Western world [1]. Therefore, an early acknowledgment of high cardiovascular risk patients is crucial to decrease the incidence of clinical events related to atherosclerosis. This could be reached through the identification of markers of subclinical arterial disease [2].

It has been previously evidenced that carotid intima-media thickness (IMT) is a noninvasive predictor of future cardiovascular disease (CVD) [3]. The increase of carotid IMT has been more commonly associated with development of coronary heart disease and stroke [4, 5]. However, even in asymptomatic subjects a slight increase of IMT may be detected by high resolution ultrasonography and may represent a marker of subclinical atherosclerosis [6]. Recently, Lorenz et al. reported a meta-analysis showing that the relative risk for stroke and myocardial infarction rises proportionally to the increase of carotid IMT [7].

The identification of risk factors associated with early atherogenesis is important to stratify the individual risk and to predict cardiovascular events [8]. It has been formerly shown a close relationship between carotid IMT and traditional cardiovascular risk factors such as ageing, hypertension, obesity, dyslipidemia, and diabetes [9]. Moreover, the presence of atherosclerotic lesions may classify high risk individuals who were not identified by the traditional risk factors evaluation. Recently, Aguilar-Shea et al., including only outpatients presenting low or intermediate cardiovascular risk according to European SCORE function, demonstrated that 18.4% of these patients were reclassified as high risk due to increased carotid IMT. The authors concluded that the measurement of carotid IMT may be an useful tool to identify asymptomatic subjects with subclinical atherosclerosis not detected by the traditional risk factors evaluation [1].

The objective of this study was to identify clinical variables associated with subclinical atherosclerosis defined by an increased carotid IMT in treated hypertensive women.

## 2. Methods

### 2.1. Study Population

We studied hypertensive women referred to Outpatient Clinics of Hypertension and Associated Metabolic Diseases at State University of Rio de Janeiro, Brazil. Participants were 40 to 65 years old, with systolic blood pressure ≥140 mmHg, and/or diastolic blood pressure ≥90 mmHg at two different times and/or in antihypertensive treatment. Patients were excluded if they had a past history of cardiovascular disease, clinically evident infection or inflammatory disease, diabetes mellitus, renal dysfunction with estimated glomerular filtration rate <60 mL/min, evidence of secondary hypertension, or using lipid-lowering medication in the last year. Vascular ultrasound was performed and the patients were divided into two groups according to the values of carotid IMT (< or ≥0.9 mm) [10]. The local Ethics Committee previously approved the study protocol and all participants gave written informed consent.

### 2.2. Clinical Evaluation

Height was measured on a clinic stadiometer. The body weight was assessed using a calibrated scale, with participants using light clothes and no shoes. The body mass index (BMI) was calculated as body weight (kg) divided by square height (m^2^). The patients were classified as eutrophic (18.5 to 24.9 Kg/m^2^), overweight (25.0 to 29.9 Kg/m^2^), or obese patients (30.0 Kg/m^2^ or more) [11].

With a nonextensible tape measure, waist circumference was measured at the midway between the lower rib and the iliac crest. Waist circumference was considered increased for women when the value was higher than 88 cm [12]. Metabolic syndrome was defined following the criteria from the Third Report of The National Cholesterol Education Program—Adult Treatment Panel III (NCEP-ATP III) [12].

Standardized questionnaires were used to obtain information about physical activity, smoking history, medication use, family history of cardiovascular disease, and duration of hypertension. Systolic and diastolic blood pressure were measured three times after a 5-minute rest, in a seated position, using an electronic device (model HEM-705CP, OMRON Healthcare Inc., IL, USA).

### 2.3. Biochemical Assay

Venous blood samples were taken after the subject had fasted for 12 hours. The serum lipids (total cholesterol, HDL-cholesterol, and triglycerides) were analyzed by colorimetry (Bioclin). The LDL-cholesterol was calculated using the Friedewald formula [13] when triglyceride value was less than 400 mg/dL. High-sensitive C-reactive protein (CRP) was determinated by turbidimetry using an automatic analyzer (BioSystems A15).

### 2.4. Carotid Ultrasound

The patient was supine with slight hyperextension and rotation of the neck in the direction opposite the probe. A linear array transducer with a multiple-frequency (7 to 12 MHz) attached to a high-resolution B-mode ultrasound system was used to acquire images by a single-sonographer blind to clinical data of subjects. Simultaneous ECG was recorded to assure the timing of end-diastolic images. Manual measurement of IMT was performed in the common carotid artery, at both sides, in a region free of plaque located approximately 20 mm from bulb. We analysed only patients without any atherosclerotic plaques. At least three values were obtained in different sites of this segment and the mean value of six measurements (three from each side) was used for analysis.

### 2.5. Statistical Analysis

Data were expressed as mean ± standard deviation or proportions when appropriate. Unpaired Student's *t-*test was used for comparison of continuous variables, with a confidence interval of 95% and *P* < 0.05 was considered statistically significant. Menopause and metabolic syndrome were considered as categorical variables and were compared by Chi-squared test. The Pearson coefficient was obtained in the correlation tests between continuous variables. Ordinal logistic regression was used to evaluate associations between increased carotid IMT and previously correlated variables. All statistical analyses were performed using SPSS statistical package (Version 18.0; Inc., Chicago, IL, USA).

## 3. Results

One hundred eighteen patients were enrolled in the study, but we excluded two patients for not having completed all assessments, resulting in 116 patients included for analysis. As expected, carotid IMT was significantly greater in the second group (0.74 ± 0.09 versus 1.03 ± 0.10 mm, *P* < 0.001). Patients in the group with greater carotid IMT were older and presented significantly higher values of systolic blood pressure and pulse pressure. The two groups had mean BMI in the overweight range and increased values of waist circumference. Regarding the biochemical profile, serum HDL-cholesterol was significantly lower, and CRP was significantly higher in the group with greater carotid IMT. Plasma glucose, creatinine, and other parameters of lipid profile were not different between the two groups (Table 1).

The proportions of smoking (21 versus 10%, *P* > 0.05), physical activity (10 versus 18%, *P* > 0.05) and menopause (65.8 versus 78.0%, *P* > 0.05) were similar between the two groups. The most used antihypertensive medications were diuretics (65.8 versus 65.7%, *P* > 0.05) and angiotensin converting enzyme inhibitors (64.5 versus 50.0%, *P* > 0.05) and there was no significant difference between the two groups.

**Figure 1:**
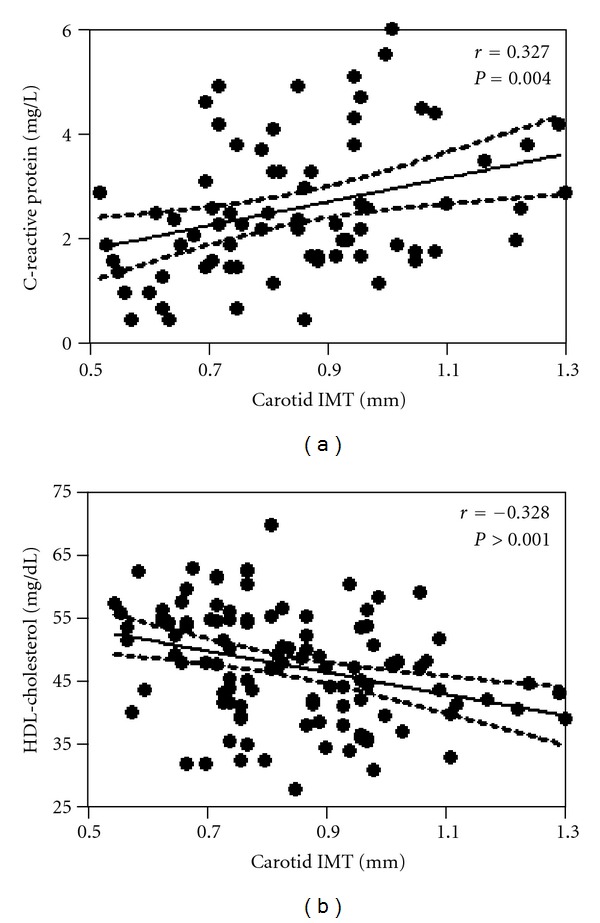
Correlation of carotid intima-media thickness (IMT) with C-reactive protein (a) and with HDL-cholesterol (b).

There was a weak but significant correlation between carotid IMT and age (*r* = 0.25, *P* = 0.007), systolic blood pressure (*r* = 0.19,  *P* = 0,009), pulse pressure (*r* = 0.30, *P* = 0.001), and LDL-cholesterol (*r* = 0.19, *P* = 0.043). Carotid IMT was also positively correlated to CRP (*r* = 0.31, *P* = 0.007) and negatively correlated to HDL-cholesterol (*r* = 0.33, *P* = 0.001) (Figures 1(a) and 1(b), resp.). To assess which variables were independently correlated with IMT, a logistic regression was performed with increased IMT as dependent variable and including age, menopause, metabolic syndrome, systolic blood pressure, pulse pressure, HDL-cholesterol and CRP as independent variables. In this model, only HDL-cholesterol, CRP, and pulse pressure were shown to be independent variables associated to increased carotid IMT (Table 2).

## 4. Discussion

Carotid IMT has been related to cardiovascular events but the link between this finding and clinical parameters in specific populations is not clear yet. In this study involving only nondiabetic treated hypertensive women with no previous cardiovascular event, carotid IMT was correlated with some clinical factors, but only pulse pressure, HDL-cholesterol, and CRP were considered independent parameters related to carotid IMT.

 It is well established that age is an important determinant factor for carotid IMT value. In a population-based study, Pastorius et al. evaluated 1448 healthy adults with no known cardiovascular disease and demonstrated that age was an independent predictor factor for increased carotid IMT [14]. Other longitudinal study including only elderly individuals was also able to show the correlation between carotid IMT values and age [15]. In contrast, age was not identified as a clinical parameter independently associated to carotid IMT in our study. This finding could be attributed to our sample study composed only by middle-age hypertensive women under drug therapy. In a recent study which included 136 subjects older than 80 years, carotid IMT was associated with age in men but not in women [16].

 Blood pressure levels have also been associated with carotid IMT. In our study, all patients were in use of antihypertensive drugs and the mean diastolic blood pressure levels were in the normal range. Systolic blood pressure was higher in the group with subclinical atherosclerosis but only pulse pressure was independently correlated with carotid IMT. In agreement with this, some studies have already reported association between increased carotid IMT and isolated systolic hypertension [14, 17, 18]. Similarly, Zanchetti et al. have also observed that ambulatory systolic blood pressure and pulse pressure were significantly correlated with carotid alterations even after adjustment for age, gender, and smoking [19].

 Several studies have already described the relationship between subclinical atherosclerosis and obesity indicated by elevated body mass index or waist circumference [20–24]. However, this association is still controversial. Tokita et al. carried out a cross-sectional study to examine the association of obesity parameters with atherosclerosis in obese patients. The authors concluded that the carotid elasticity, but not IMT, was the only variable that reflects visceral fat accumulation in those obese subjects [25]. In the present study, there was no difference between the two groups in relation to anthropometric parameters. Despite an increased waist circumference in both groups, the mean value was not statistically different and there was no significant correlation with carotid IMT. Nevertheless, this finding could be justified by the mean body mass index value in the overweight range in both groups with few patients presenting normal body mass index. Although metabolic syndrome was significantly more frequent in the group with increased carotid IMT, this factor was not independently associated with subclinical atherosclerosis in this study population after logistic regression.

The protective effect of HDL-cholesterol is already well debated and it is not clarified yet. A recent meta-analysis reported that the great majority of the included studies observed an inverse association between HDL-cholesterol and carotid atherosclerosis [26]. However, six longitudinal studies in this systematic review demonstrated conflicting results, with three reports showing no relationship and an evidence of a weak association. Fan and Dwyer observed the progression of carotid IMT in a cohort study that enrolled 500 individuals aged 40–60 years. In the beginning of this study, carotid IMT was inversely associated with serum levels of HDL-cholesterol in men and in women. Nevertheless, after an adjustment for some potential confounding factors, the progression of carotid IMT was negatively associated with HDL-cholesterol in men, but directly in women. The authors suggest that the antiatherogenic effects of HDL-cholesterol could be attenuated in middle-aged women [27]. The lack of association between carotid atherosclerosis and HDL-cholesterol was also observed in a recent population-based study [14]. In the current study, both groups presented low values of HDL-cholesterol in relation to those expected for women, but significantly lower in patients with increased carotid IMT. This result indicates that even small increases of HDL-cholesterol could be protective against subclinical atherosclerosis in hypertensive women. Other plasma lipids were not different between the two groups in the current study. On the other hand, the same authors have previously shown a relationship between carotid IMT and serum triglycerides, total cholesterol and LDL-cholesterol [28, 29].

Elevated CRP has been considered a predictor of cardiovascular events. A clinical history carefully obtained and a complete physical examination are needed in order to rule out an obvious cause for increased CRP [30]. In the current study, patients presenting infections or inflammatory diseases were excluded to avoid interpretation errors on CRP analysis. High-sensitive CRP levels were significantly higher in the group with higher carotid IMT values. However, even in the group with lower carotid IMT, CRP levels corresponded to intermediate cardiovascular risk. A possible explanation for this finding is because all patients were hypertensive with increased abdominal circumference which is also related to raised CRP levels [31, 32]. Increased CRP levels without intima-media carotid artery thickening may suggest a possible role as a risk marker of cardiovascular disease and a mediator in atherogenesis due to its effects in endothelial cells as previously reported [6]. In addition, CRP levels were independently associated with increased IMT as it was previously found by other studies, [33, 34] including Framingham cohort [35]. Nevertheless, these findings were not confirmed by all studies [36, 37]. Indeed, the relationship between carotid atherosclerosis and CRP levels were not significant after adjustment for other risk factors in some studies [38, 39].

## 5. Conclusion

 We concluded that pulse pressure, HDL-cholesterol, and high-sensitive CRP are variables correlated with carotid IMT in treated hypertensive women with no diabetes or previous cardiovascular events. Therefore, these parameters should be carefully followed in this population as potential markers of subclinical atherosclerosis. Noticeably, further studies are needed to establish the prognostic value of clinical and biochemical factors associated with increased carotid IMT in treated hypertensive patients.

## Figures and Tables

**Table 1:** Clinical, anthropometric and biochemical characteristics of both groups.

Parameter	IMT ≤ 0.9 (*n* = 76)	IMT > 0.9 (*n* = 40)	*P-*value
Age, years	50.8 ± 6.6	53.5 ± 7.1*	0.044
BMI, kg/m^2^	28.9 ± 4.5	29.5 ± 4.9	0.471
Waist, cm	98.4 ± 11.2	99.1 ± 11.1	0.733
Systolic BP, mmHg	135 ± 16	143 ± 21*	0.020
Diastolic BP, mmHg	85 ± 11	83 ± 9	0.253
Pulse pressure, mmHg	49 ± 11	60 ± 17***	<0.001
Metabolic Syndrome, *n*(%)	49(64.5)	33(82.5)*	0.043
eGFR, mL/min	75 ± 14	74 ± 15	0.863
Glucose, mg/dL	88 ± 19	87 ± 14	0.735
Creatinine, mg/dL	0.80 ± 0.13	0.81 ± 0.13	0.740
Total cholesterol, mg/dL	209 ± 54	255 ± 64	0.157
HDL-cholesterol, mg/dL	49 ± 8	44 ± 7**	0.004
LDL-cholesterol, mg/dL	130 ± 50	149 ± 57	0.070
Triglycerides, mg/dL	144 ± 84	150 ± 71	0.731
hsCRP, mg/L	2.31 ± 1.21	3.05 ± 1.34*	0.016
IMT, mm	0.74 ± 0.09	1.03 ± 0.10***	<0.001

Data expressed as mean ± SD or *n*(%) when indicated; IMT: intima-media thickness; BMI: body mass index; BP: blood pressure; eGFR: estimated glomerular filtration rate; HDL: high density lipoprotein; LDL: low density lipoprotein; hsCRP: high-sensitive C-reactive protein; ACE: angiotensin converting enzyme; ARB: angiotensin receptor blocker. *P < 0.05, **P < 0.01***, *P* < 0.001.

**Table 2:** Logistic regression model for increased carotid intima-media thickness (IMT > 0.9 mm) as dependent variable.

Variables	*β*	Standard error	*P *value
HDL-cholesterol	1.126	0.051	0.019
hsCRP	0.448	0.293	0.006
Pulse pressure	0.917	0.046	0.049

Variables included in the initial model: age, menopause, metabolic syndrome, systolic blood pressure, pulse pressure, HDL (high density lipoprotein)-cholesterol, and high-sensitive C-reactive protein (hsCRP).
